# Effects of Straw Addition on Soil Priming Effects Under Different Tillage and Straw Return Modes

**DOI:** 10.3390/plants13223188

**Published:** 2024-11-13

**Authors:** Peixuan Cai, Haixia Wang, Zhihui Zhao, Xue Li, Ying Wang, Xiumei Zhan, Xiaori Han

**Affiliations:** 1 College of Land and Environment, Shenyang Agricultural University, Shenyang 110866, China; cpx18642985383@163.com (P.C.); 13384328675@163.com (H.W.); zhaozhihui021@163.com (Z.Z.); 2024500003@syau.edu.cn (X.L.); 2 Liaoning Agricultural Development Center, Shenyang 110034, China; xyxk20052008@163.com

**Keywords:** straw return mode, soil organic carbon, labile organic carbon, priming effect

## Abstract

This study aims to investigate the impact of straw addition on soil activation effects under different tillage practices, providing a scientific basis for establishing reasonable straw return measures in the southern Northeast Plain, thus enhancing soil fertility, and mitigating greenhouse effects. Soil samples were collected from various straw return practices that were conducted continuously for two years as follows: rotary tillage without straw return (RTO), deep tillage combined with straw incorporation (PT), rotary tillage with straw incorporation (RT), and no-till with straw cover (NT). The samples were incubated in the dark at 25 °C for 70 days. We measured the CO_2_ release rate and cumulative release, apparent activation effect, soil organic carbon, active microbial biomass organic carbon, soluble organic carbon, and easily oxidizable organic carbon to clarify the effects of straw addition on soil activation under different tillage practices. The results indicate that a straw addition promotes the mineralization of soil organic carbon while also increasing the content of active organic carbon components. The CO_2_ release rates and cumulative release under different tillage practices were as follows: PT > NT > RT. The contents of the active microbial biomass organic carbon, soluble organic carbon, and easily oxidizable organic carbon increased by 16.62% to 131.88%, 4.36% to 57.59%, and 12.10% to 57.97%, respectively, compared to the control without the straw addition. Except for the RT practice, the addition of straw significantly enhanced the instability of soil organic carbon in the PT, NT, and RTO practices, with increases of 51.75%, 48.29%, and 27.90%, respectively. Different straw return practices altered the physical and chemical properties of the soil, resulting in significant differences in the strength of the apparent activation effect. Notably, the apparent activation effect of RT was reduced by 86.42% compared to RTO, while that of NT was reduced by 36.99% compared to PT. A highly significant positive correlation was observed between the apparent activation effect and the unstable carbon components in the soil, indicating that higher levels of easily decomposable organic carbon corresponded to stronger apparent activation effects. In conclusion, it is recommended that in this region, rotary tillage should be adopted for straw return in the first 2 to 3 years, as this practice is beneficial for the formation and stabilization of organic carbon in the short term. As the duration of straw return increases, adjustments can be made based on the degree of soil organic carbon retention and soil fertility status.

## 1. Introduction

The soil priming effect refers to the significant changes in short-term turnover of soil organic matter caused by the addition of various organic substances [1]. The magnitude of the overall priming effect is determined by the combined contributions of the apparent priming effect and true priming effect [2,3,4,5]. The true priming effect arises from the additional decomposition of recalcitrant organic matter, while the apparent priming effect arises from the increased microbial carbon turnover, both of which are controlled by microbial activity. Soil microorganisms assimilate plant-derived carbon substrates through assimilation to synthesize their own biomass, and through microbial biomass accumulation, microbial-derived organic carbon is transferred to the soil [6]. Crop straw is an important external supplement for the soil organic carbon pool in agricultural soils [7], and its incorporation into the soil can alter the mineralization process of the original soil organic matter, thus inducing the priming effect and affecting the soil’s carbon balance and turnover. Studies have shown that long-term straw incorporation may increase the SOC content, but it can also have little or even negative effects [8,9,10]. This mainly depends on the intensity and direction of the priming effect. When the newly added organic matter promotes the mineralization of the existing organic matter in the soil, a “positive priming effect” occurs. In this case, if the amount of SOC sequestration is higher than the amount of SOC mineralization loss, the SOC content tends to increase. Conversely, if it inhibits the mineralization of the existing organic matter in the soil, a “negative priming effect” occurs, which favors the accumulation of soil organic carbon [11,12,13].

Studies have found that tillage practices and crop straw incorporation are important factors in influencing the intensity and direction of the priming effect in agricultural systems. Active organic carbon in the soil accounts for 1% to 5% of the total soil organic carbon (SOC) [14]. Due to its short turnover time and sensitivity to external organic carbon, active organic carbon plays a crucial role in a series of decomposition and transformation processes [15]. Soil microbial biomass carbon (MBC), an important indicator for assessing soil microbial biomass, significantly reflects the quantity and activity of soil microorganisms [16]. Numerous experiments with substantial straw addition have indicated a close relationship between straw and the soil’s microbial carbon content, as straw provides the ample energy necessary for microbial activity, facilitating the proliferation of soil microorganisms [17]. Easily oxidizable organic carbon (EOC) can be readily decomposed and mineralized by soil microorganisms, serving as a nutrient source for plants. Dissolved organic carbon (DOC) is a smaller, active carbon component of soil organic carbon, characterized by its instability and easy availability to microorganisms, often released into the atmosphere as CO_2_ [18]. It plays an important role as a nutrient transport carrier [19,20]. Currently, there is a substantial amount of research on the priming effect and the active organic carbon fractions in response to tillage practices and straw incorporation. Kan et al. found that under no-tillage conditions, straw incorporation reduced organic carbon mineralization and the intensity of the priming effect [21]. This was attributed to soil compaction and reduced aeration in no-till soils, which affected the aerobic microbial community and resulted in a decrease in the intensity of the priming effect [22]. In comparison to no-tillage, deep tillage with straw incorporation improved the effectiveness of the soil’s active carbon pool, promoted bacterial growth and activity, and accelerated the microbial decomposition and priming of organic carbon [23]. Similarly, conventional till age disrupts soil aggregates, protecting the organic carbon within aggregates that are more exposed and thus increasing the availability of aggregate-associated organic carbon fractions to microorganisms. This affects the DOC content in the tillage layer and is accompanied by an enhanced priming effect [24]. Jenkinson suggested that unstable carbon pools are more susceptible to the priming effect compared to stable carbon pools [25]. The stoichiometric theory indicates that microbial activity is maximally stimulated when the nutrient ratios are more balanced [26]. The priming effect is primarily controlled by nutrient availability rather than tillage practices. Although tillage can cause significant changes in soil organic carbon and microbial biomass, the ultimate effect of tillage practices on the intensity of the priming effect is mediated by the ratio of active organic carbon to nutrient content in the soil [27]. Therefore, studying the effects of different tillage practices and straw incorporation methods on the priming effect, soil organic carbon, and its active fractions is beneficial for improving farmland quality and achieving a more efficient utilization of local straw resources [17].

The Northeast Plain is characterized by vast territory and significant variations in climatic conditions [28]. It has relatively abundant water and heat conditions, and maize is one of the main cereal crops grown in the region, with high maize straw production [29]. Currently, common methods of straw incorporation include straw burial, straw mulching, and straw incorporation with stubble, combined with corresponding cultivation techniques such as rotary tillage with straw burial, deep loosening with straw burial, and no-tillage with straw mulching [30,31]. Different climatic and soil conditions in different regions result in varying effects of different straw incorporation methods on the mineralization and sequestration characteristics of soil organic carbon [32]. This directly affects improvements in farmland quality and the widespread application of straw incorporation techniques [33]. Due to the lack of comparative studies on straw incorporation techniques tailored to local climatic and soil conditions and the priming effect of soil organic carbon, the utilization rate of straw incorporation has remained low in practical production [34,35]. Based on specific soil and climatic conditions, this experiment conducted cultivation trials to investigate the mineralization characteristics of soil organic carbon and the changes in the active carbon pool fractions after the addition of straw under different straw incorporation methods. It aims to reveal the mechanisms of the priming effect of soil under different straw incorporation methods and provide theoretical support and technical references for optimizing straw incorporation techniques in the southern part of the Northeast Plain. The goal is to achieve an efficient utilization of straw resources, sustainable farmland carbon storage, and environmentally friendly agricultural practices, ultimately promoting soil fertility.

## 2. Materials and Methods

### 2.1. Experimental Description

The field experiment on maize was conducted in Dong Sifangtai Town, Haicheng City, Liaoning Province, China, from 2019 to 2020. The experimental site is located in the southern part of Liaoning Province (40°48′ N, 122°37′ E) and has an average annual temperature of around 10.4 °C. The accumulated temperature ranges from 3000 to 3100 °C annually, with a precipitation of 600–800 mm and a frost-free period of approximately 170 days. The region has a warm, temperate continental monsoon climate. Prior to the experiment, the field had been under continuous rotary tillage, and straw had not been incorporated. The soil type is classified as brown soil, and the basic physicochemical properties of the 0–20 cm plow layer soil were as follows: an organic carbon content of 12.94 g·kg^−1^, total nitrogen content of 0.82 g·kg^−1^, alkaline hydrolyzable nitrogen content of 129.60 mg·kg^−1^, available phosphorus content of 25.20 mg·kg^−1^, available potassium content of 121.94 g·kg^−1^, and a pH value of 5.21.

The experiment consisted of four treatments: rotary tillage without straw incorporation (RTO), deep loosening with deep rotary tillage and straw incorporation (PT), rotary tillage with straw incorporation (RT), and no-tillage with straw mulching (NT). The crop planted was maize. After the maize harvests in 2018 and 2019, the straw was directly crushed using a combine harvester and evenly spread on the soil’s surface. In the RTO treatment, the crushed straw was collected and removed from the field. The field was then plowed to a depth of 15–20 cm using a disc plow, followed by land preparation. In the RT treatment, the crushed straw was mixed with the soil under the action of the disc plow. In the PT treatment, deep loosening was performed using a subsoiler to a depth of 30–35 cm, followed by deep rotary tillage to a depth of 20–25 cm, during which the straw was mixed with the soil. The NT treatment involved no specific treatment, and direct seeding was conducted using a no-till seeder in the spring of 2019 and 2020. Each treatment covered an area of 6670 m^2^, and no replicates were set. Fertilization and field management practices were consistent with local methods.

### 2.2. Experimental Material

The soil samples for the experiment were collected from the 0–20 cm soil layer of each treatment after the 2020 corn harvest, using a multi-point sampling method. The soil samples were divided into two parts, with one part used for determining the basic physical and chemical properties of the soil and the other part sieved through a 4 mm sieve for indoor cultivation experiments. The basic physical and chemical properties of the soil samples are shown in Table 1.

The straw used for cultivation was taken from the same experimental treatments after the 2020 corn harvest, dried to a constant weight at 60 °C, crushed, sieved through a 1 mm sieve, and stored in sealed bags for later use. The carbon content of the corn straw was 444.57 g·kg^−1^, and the nitrogen content was 12.51 g·kg^−1^.

### 2.3. Incubation Experiment

Indoor constant temperature cultivation experiments were set up with two levels of straw addition: no addition of straw (M0) and addition of straw (M1), forming a total of 8 treatments. These treatments included no straw added to the soil after rotary tillage (RTO + M0), no straw added to the deep tillage soil (PT + M0), no straw added to the rotary tillage soil (RT + M0), no straw added to the no-tillage soil (NT + M0), straw added to the soil after rotary tillage (RTO + M1), straw added to the deep tillage soil (PT + M1), straw added to the rotary tillage soil (RT + M1), and straw added to the no-tillage soil (NT + M1). Each treatment was replicated 12 times (destructive sampling was performed to determine the soil’s active organic carbon). The amount of corn straw added in the treatments with the straw addition was 10 g·kg^−1^ (making the mass of air-dried corn straw 1% of the mass of air-dried soil). Before formal cultivation, the soil moisture was adjusted to 40% of the field capacity, and pre-cultivation was conducted in the dark at 25 °C for 7 days to restore microbial activity and function [1].

Seventy grams of soil (dry weight) were placed in 600 mL culture bottles, and corn straw was thoroughly mixed with the soil according to the different treatments. Distilled water was used to adjust the soil moisture to 60% of the field capacity. Additionally, culture bottles without soil and straw were set up as controls, sealed, and placed in a constant temperature incubator at 25 °C in a random arrangement under dark conditions for 70 days. Gas produced in the culture bottles was collected on days 1, 3, 5, 7, 11, 15, 22, 40, and 70 after cultivation. After each gas collection, the culture bottles were ventilated for 30 min to ensure a thorough exchange of gas with the atmosphere. The soil moisture was adjusted using a weighing method to maintain it at 60% of the field capacity.

### 2.4. Soil Sampling and Analysis

Before each gas collection, the syringe was purged with air and then inserted into the three-way valve for gas collection from the culture bottles on days 1, 3, 5, 7, 11, 15, 22, 40, and 70 after cultivation. Gas was collected twice using a 50 mL syringe, with a total of 60 mL of gas stored in a 100 mL self-sealing bag for the subsequent measurement of CO_2_ content using a gas chromatograph.

Soil samples were collected on days 0, 7, 15, and 70 for analyses of active microbial biomass carbon (MBC) and dissolved organic carbon (DOC). After the cultivation period, the soil organic carbon (SOC), easily oxidizable organic carbon (EOC), and extractable organic carbon were also determined.

Air-dried soil samples were ground and sieved through a 100-mesh sieve. Approximately 30–40 mg of the soil sample was weighed in a tared tin boat on an electronic balance (accuracy: one millionth). The air was removed, and the SOC content was determined using an elemental analyzer (Elementar Vario EL III, Detmold, Germany). The soil MBC content was determined using the chloroform fumigation-extraction method with K_2_SO_4_ [36]. Two fresh soil samples, each equivalent to 10 g of dry soil weight, were weighed. One sample was placed in a vacuum desiccator containing 50 mL of anhydrous chloroform and dried under vacuum for 24 h. After removing the chloroform, the other sample was extracted with 0.5 mol·L^−1^ K_2_SO_4_ (soil-to-water ratio of 1:4) under the same conditions. The extract was then analyzed using a total organic carbon analyzer (Multi N/C3100, Dresden, Germany). The MBC was calculated as the difference in organic carbon content between the fumigated and non-fumigated soil extracts divided by a factor of 0.45. The non-fumigated soil samples were used to determine the DOC content. The EOC content in the soil was determined using a 333 mmol·L^−1^ KMnO_4_ solution oxidation method. In a centrifuge tube, 15 mg of the soil sample was mixed with 25 mL of the 333 mmol·L^−1^ KMnO_4_ solution [37]. After centrifugation at 4000 rpm for 5 min, the supernatant was diluted 250 times with water, and the color was measured at a wavelength of 565 nm using a spectrophotometer. The calculation method was based on the loss of 1 mmol of KMnO_4_ solution, being equivalent to the oxidation of 9 mg of carbon.

### 2.5. Calculation Methods

Soil organic carbon mineralization refers to the process in which organic carbon-containing substances present in the soil undergo transformation into inorganic forms under the action of microorganisms, releasing CO_2_. The rate of mineralization is reflected by the rate of CO_2_ emission (mg·kg^−1^·d^−1^) with the following formulas:

(1) $${CO}_{2}\left(f\right)=\frac{(C_{1}-C_{0})\;\times \;M\;\times \;V\;\times \;10^{3}}{m\;\times \;MVcorr\;\times \;t}$$

where C_1_ is the CO_2_ concentration in the gas-collecting bottle during gas sampling, C_0_ is the CO_2_ concentration in the bottle without soil and straw, M represents the molecular weight of CO_2_ (44 g·mol^−1^), V represents the headspace volume of the bottle (m^3^), m represents the dry weight of soil (kg), and MVcorr is the temperature-corrected gas molar volume (0.0242 m^3^·mol^−1^).

The apparent priming effect (PE) is calculated using the formula from previous research [38,39] as follows:

(2) $$PE=\frac{{CO}_{2}\text{-}C_{treatment}-{CO}_{2}\text{-}C_{control}}{{CO}_{2}\text{-}C_{control}}\times 100$$

where CO_2_-C_treatment_ refers to the soil CO_2_ emission after the addition of straw, while CO_2_-C_control_ represents the soil CO_2_ emission without the addition of straw; a positive excitation effect occurs when CO_2_-C_treatment_ > CO_2_-C_control_, and conversely, a negative excitation effect occurs when CO_2_-C_treatment_ < CO_2_-C_control_.

The calculation formula for the soil organic carbon instability (LA) is as follows:

(3) LA=EOC/(SOC−EOC)

where EOC is the content of easily oxidizable organic carbon in the soil (g·kg^−1^), and SOC is the content of soil organic carbon (g·kg^−1^).

### 2.6. Data Analysis

In this study, Excel 2023 was used for the statistical calculations. SPSS 23.0 software was employed to conduct a two-way ANOVA on CO_2_’s cumulative emissions, SOC, and active components concerning tillage practices, straw addition, and their interaction. Dunnett’s multiple comparison method was applied for the significance analysis (*p* < 0.05) of the apparent activation effects, soil organic carbon, and its active components under different tillage practices with the straw addition. Pearson correlation analysis was performed to assess the relationships between the factors influencing the activation effects. Data normalization for different treatment samples was carried out using SPSS 23.0, and graphs were generated using Origin 2021.

## 3. Results

### 3.1. Effect of Different Tillage Modes on Soil Physicochemical Property

According to Table 1, it can be observed that after two years of continuous straw returning under different tillage practices, there was a significant increase in the total nitrogen and available potassium contents. However, there was a significant decrease in the soil alkali-hydrolyzable nitrogen content (*p* < 0.05, *n* = 3). Compared to the initial state, the SOC content had decreased by 0.42% and 3.44% in the RTO and NT modes, respectively, while in the RT and PT modes, the SOC content had increased by 2.04% and 8.99%, respectively. In the PT mode, the soil organic carbon, total nitrogen, and available potassium contents were the highest, while the NT mode had the lowest organic carbon content. There was no significant difference in total nitrogen and the available potassium content among the RTO, RT, and NT modes. The RT mode had the highest available phosphorus content, followed by the NT mode, with no significant difference between the RTO and PT modes. The pH values of the RTO, RT, and NT modes had significantly increased compared to the initial experiment, with increases of 9.34%, 2.69%, and 4.74%, respectively. Although the pH in the PT mode had decreased compared to the initial experiment, the difference was not significant. In comparison to the RTO mode, MBC and DOC under the straw returning treatment had increased by 8.03–15.79% and 7.76–32.99%, respectively. Compared to the RTO mode, the PT mode showed a significant increase in the EOC content (56.06%) and LA (52.92%), while the NT mode showed an increase of 11.62% and 17.37%, respectively. The RT mode had a slight decrease in the EOC (−5.56%) and LA (−8.90%), but the difference from RTO was not significant. It was evident that different cultivation practices over two years had altered the soil’s physicochemical properties, and the response of the soil’s physicochemical properties varied with different cultivation and straw returning modes.

### 3.2. Effect of Straw Addition on Soil Organic Carbon Mineralization and the Apparent Priming Effect

#### 3.2.1. Effect on the Rate of Soil CO_2_ Emission and Its Cumulative Emission

**Figure 1:**
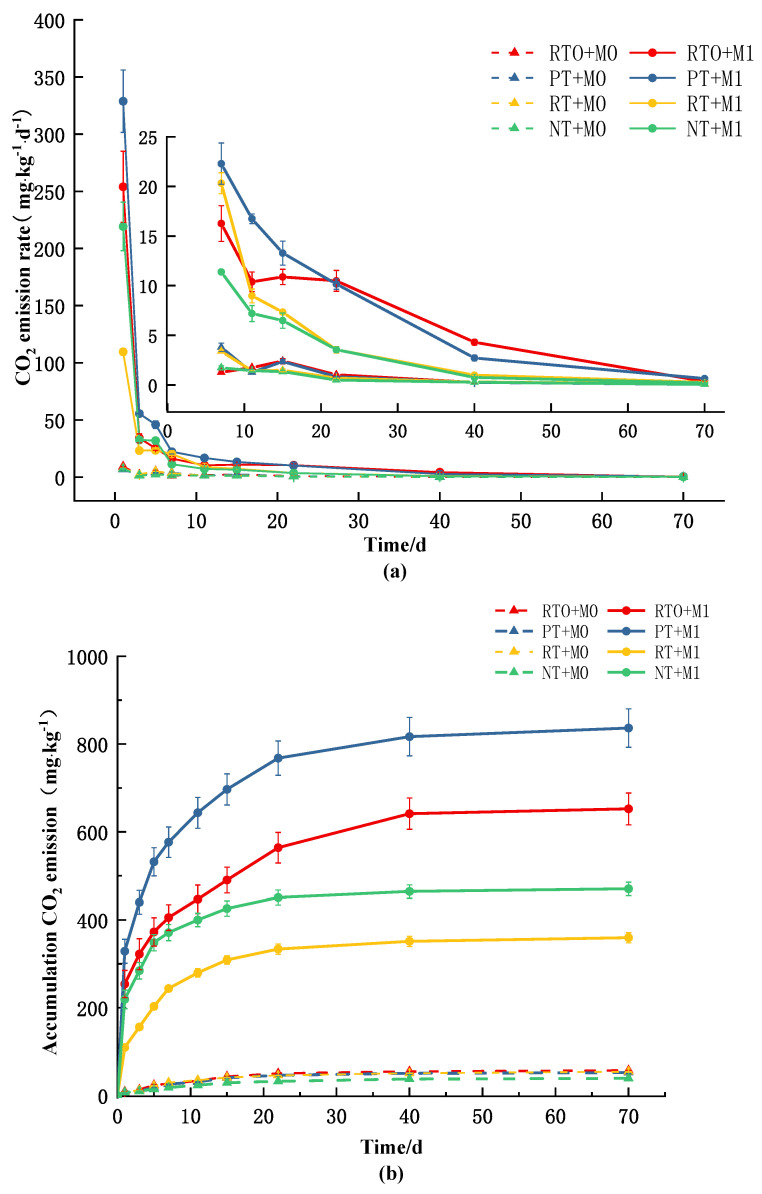
The CO_2_ emission rate (**a**) and accumulation of CO_2_ emissions (**b**) in soils under different treatments. (*p* < 0.05, *n* = 3). The inserted figure in the CO_2_ emission rate (**a**) graph represents the dynamic changes in soil CO_2_ emission rates during the cultivation period from 7–70 days. The error bars represent the standard deviation (SD).

Based on Figure 1, it is evident that throughout the entire cultivation period, regardless of the addition of straw, the CO_2_ emission rates for all treatments exhibited a gradual decrease over time. The overall trend can be divided into three periods: 0–3 days as the first period of rapid decline, 3–40 days as the second period of fluctuating decline, and 40–70 days as the third period of stabilized mineralization rates. Additionally, a peak in CO_2_ emission rates occurred on the first day of the experiment for all treatments. Under the same cultivation practices, the CO_2_ emission rates for treatments with added straw were significantly higher than those without straw. In the absence of straw, the RTO mode exhibited the highest CO_2_ emission rate, while the PT mode showed the lowest. However, with the addition of straw, this trend was altered, with the PT mode displaying the highest CO_2_ emission rate and the RT mode exhibiting the lowest.

The results of a two-factor analysis of variance indicated that the cumulative soil CO_2_ emissions were significantly influenced by both the soil tillage mode and the addition of straw, as well as their interaction (*p* < 0.001). Regardless of whether straw was added, the cumulative soil CO_2_ emissions showed a continuously increasing trend over time. In the absence of straw (M0), the cumulative soil CO_2_ emissions were associated with the tillage mode. The RTO mode exhibited the highest CO_2_ cumulative emissions, followed by PT and RT, with no significant difference between the two tillage modes. The NT mode had the lowest cumulative CO_2_ emissions. After adding an equal amount of straw (M1), the PT mode showed significantly higher cumulative CO_2_ emissions compared to other treatments, while the RT mode had the lowest cumulative CO_2_ emissions. The cumulative CO_2_ emissions in the RT mode decreased by 81.45% compared to the RTO mode, and the NT mode decreased by 77.81% compared to the PT mode. Compared to the treatments without straw addition, the addition of straw significantly increased the cumulative soil CO_2_ emissions for all treatments. The increase in CO_2_ emissions varied under different tillage practices after adding straw, with the PT mode showing the greatest increase and the RT mode showing the smallest increase. This suggests that the addition of straw enhances soil respiration, with the PT mode having the strongest promoting effect on soil respiration, followed by the NT mode, and the weakest effect in the RT mode.

#### 3.2.2. Apparent Priming Effect

**Figure 2:**
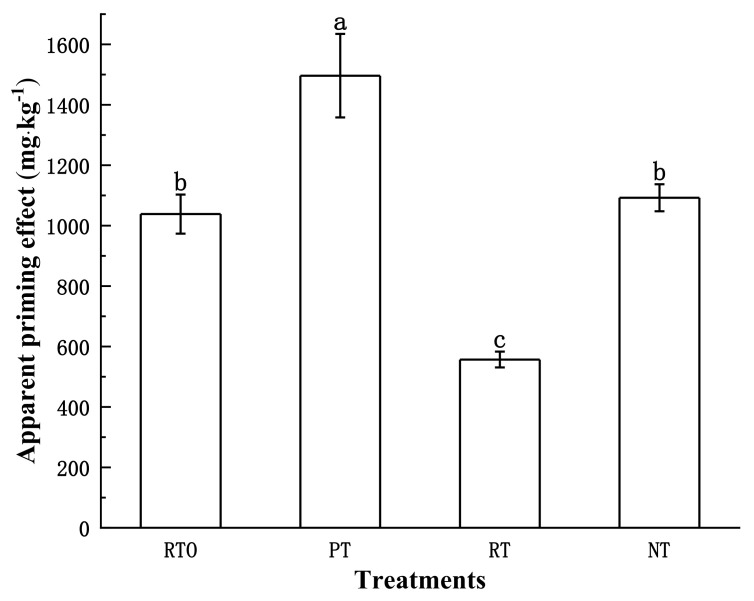
Apparent priming effect under different treatments. The error bars represent the standard deviation (SD). The significant differences between treatments are indicated by different lowercase letters labeled. (*p* < 0.05, *n* = 3).

Based on Figure 2, it can be observed that the addition of straw promotes the mineralization of soil organic carbon under different tillage modes. The apparent priming effect (PE) in each treatment exhibited a positive priming effect, and there were significant differences among various tillage and residue management modes (*p* < 0.05, *n* = 3). The order of PE strength was PT > NT > RTO > RT, indicating that the PE in the PT mode was significantly higher than in the other three treatments. The RT mode had the lowest PE, and there were no significant differences between NT and RTO. Compared to the RTO mode, the PE in the RT mode decreased by 86.42%, while compared to the PT mode, the PE in the NT mode decreased by 36.99%.

### 3.3. Effect of Straw Addition on Soil Organic Carbon and Its Active Components Under Different Tillage Modes

#### 3.3.1. Soil Organic Carbon

The results of the two-factor analysis of variance (Table 2) indicated that the SOC content was significantly influenced by the main effects of the soil tillage mode, straw addition level, and their significant interaction (*p* < 0.001). From Table 2, it can be observed that after 70 days of cultivation, in the treatments without straw addition, the PT mode exhibited the highest SOC content, reaching 15.35 g·kg^−1^. This was significantly higher than the other three tillage modes, with increases of 2.06%, 2.54%, and 5.43% compared to the RTO, RT, and NT modes, respectively. The SOC content in the RT and RTO modes followed at 14.97 g·kg^−1^ and 15.04 g·kg^−1^, respectively, but the difference was not significant. The NT mode had the lowest SOC content at 14.56 g·kg^−1^. Under the conditions of straw addition, significant differences were observed among the treatments. The SOC content in the RT mode was the highest, being 5.30% higher than the RTO mode, while the NT mode had the lowest SOC content, being 5.80% lower than the PT mode.

The addition of straw significantly increased the SOC content in all treatments, but the enhancement effect varied under different tillage modes. After cultivation, compared to the treatments without straw addition, the SOC content in the RT, PT, NT, and RTO modes increased by 14.09%, 8.86%, 8.10%, and 7.85%, respectively. Compared to before cultivation, the RT mode exhibited the greatest increase in SOC content after the straw addition, with an increase of 17.85%, followed by the NT and RTO modes, which increased by 14.47% and 14.39%, respectively. The PT mode showed the smallest increase in SOC content, at 7.67%. In summary, adding straw to the soil is beneficial for increasing the SOC content, but the enhancement effect varies under different tillage modes. In this experiment, the addition of straw was more conducive to the accumulation of soil organic carbon in the rotary tillage mode.

#### 3.3.2. Soil Microbial Biomass Carbon (MBC) Content

**Figure 3:**
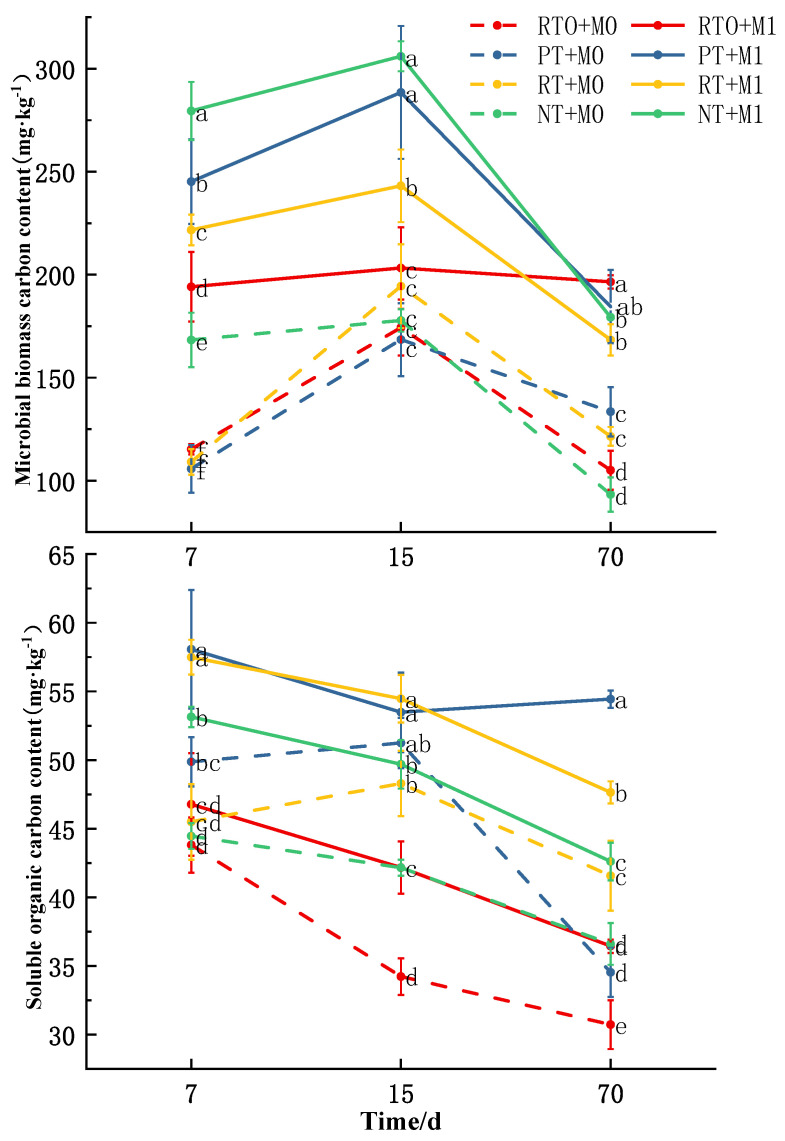
Changes in microbial biomass carbon content and soluble organic carbon content in different times and treatments. The error bars represent the standard deviation (SD). The significant differences between treatments are indicated by different lowercase letters labeled (*p* < 0.05, *n* = 3).

The results of the analysis of variance (Table 3) revealed that the soil MBC content was significantly influenced by the tillage mode, straw addition level, cultivation time, and their interactions (*p* < 0.001). As depicted in Figure 3, throughout the entire cultivation period, the overall trend of the soil MBC content showed an initial increase followed by a subsequent decrease. In all treatments, the soil MBC content reached its maximum on the 15th day of cultivation and thereafter decreased with prolonged cultivation time. The addition of straw increased the soil MBC content compared to treatments without straw, with all differences reaching a significant level except on the 15th day of cultivation under the RTO mode.

The impact of the straw addition on the MBC content varied under different tillage modes. After adding straw, compared to the treatments without straw, the RTO, PT, RT, and NT modes exhibited increases ranging from 16.62–87.04%, 38.24–131.88%, 25.05–103.27%, and 66.06–92.20%, respectively, at various time points. On the 7th day of cultivation, the MBC content differed significantly among treatments, with the NT mode having a significantly higher MBC content than the other three treatments, regardless of the straw addition. After adding straw, the RTO mode exhibited the lowest MBC content. On the 15th day of cultivation, without the straw addition, there was no significant difference in the MBC content among the four tillage modes. However, the addition of straw significantly altered this distribution, with the order of the MBC content among treatments being NT > PT > RT > RTO, with no significant difference between NT and PT. By the 70th day of cultivation, the changing trend among treatments shifted, and after adding straw, the order of the MBC content became RTO > PT > NT > RT. The RTO mode showed a significant increase of 16.70% compared to the RT mode, while the PT mode only increased the MBC content by 2.90% compared to the NT mode, with no significant difference.

Throughout the entire cultivation period, the MBC content in the RTO treatment did not exhibit significant increases or decreases, and the change in RT mode was relatively gradual. In contrast, the MBC content fluctuated most dramatically in the NT and PT modes.

#### 3.3.3. Soil Dissolved Organic Carbon (DOC) Content

The results of the analysis of variance (Table 3) indicated that the soil DOC content was significantly or extremely significantly affected by the tillage mode, straw addition level, cultivation time, and their interactions. As illustrated in Figure 3, the soil DOC content in all four tillage modes significantly increased after the straw addition. Throughout the cultivation period, there was a general trend of gradual decline in DOC content across all treatments as the cultivation time increased.

The impact of the straw addition on the DOC content varied under different tillage modes. After adding straw, compared to the treatments without straw, the RTO, PT, RT, and NT modes exhibited increases ranging from 6.75–23.18%, 4.36–57.59%, 12.78–26.36%, and 16.41–19.52%, respectively, at various time points. On the 7th day of cultivation, regardless of the straw addition, the trend in DOC content change was PT > RT > NT > RTO. On the 15th day of cultivation, for the treatments with a straw addition, the trend in DOC content changed to RT > PT > NT > RTO but without a significant difference between PT and RT. As the cultivation experiment progressed, except for the PT + M1 treatment, the DOC content in all other treatments gradually decreased. By the 70th day of cultivation, the differences in DOC content after the straw addition among the four tillage modes were significant, still showing the order of PT > RT > NT > RTO. The DOC content in the PT treatment was significantly higher than that in the other treatments, with an increase of 30.72% compared to the RT mode and 27.74% compared to the NT mode.

#### 3.3.4. Soil Easily Oxidized Organic Carbon (EOC) Content

**Figure 4:**
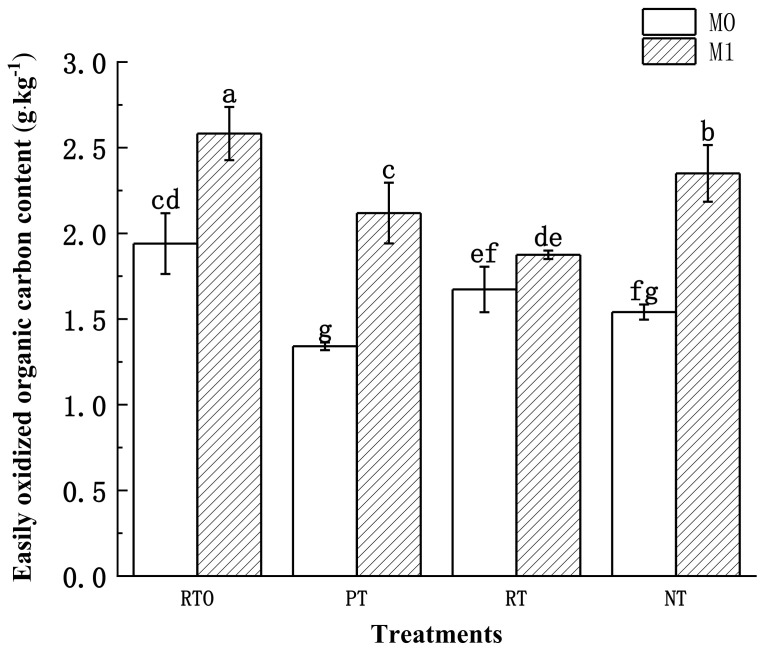
Content of easily oxidized organic carbon under different treatments. The error bars represent the standard deviation (SD). The significant differences between treatments are indicated by different lowercase letters labeled (*p* < 0.05, *n* = 3).

The results of the two-factor analysis of variance (Table 4) indicated that the soil EOC was significantly influenced by the main effects of the soil tillage mode and straw addition level, as well as their significant interaction. As observed in Figure 4, after 70 days of cultivation, there were significant differences in the EOC content among different tillage and residue management modes, ranging from 1.34–2.58 g·kg^−1^. At the end of the cultivation, without the straw addition, the trend in EOC content change among the treatments was RTO > RT > NT > PT. After adding straw, the trend shifted to RTO > NT > PT > RT. Additionally, the addition of straw significantly increased the soil EOC content in all treatments compared to those without straw. Among them, the PT mode showed the greatest increase, with a significant enhancement of 57.97%. Following that, the NT and RTO modes exhibited significant increases of 52.53% and 33.10%, respectively, while the RT mode showed the smallest increase of 12.10%, and the enhancement effect was not significant.

Compared to before cultivation, the treatments without the straw addition showed a decrease in the EOC content. Among them, the PT mode exhibited the greatest decline, with a reduction of 56.66%. The NT and RT modes followed, with reductions of 30.22% and 10.60%, respectively. The RTO mode showed the smallest decline, with a reduction of 2.17%. In contrast, the treatments with straw addition, except for PT, showed an increase in the EOC content. Among them, the RTO mode exhibited the largest increase, rising by 30.22%. The NT mode followed with an increase of 6.43%, while the RT mode showed the smallest increase of 0.21%, and the enhancement effect was not significant.

#### 3.3.5. Soil Organic Carbon Instability (LA)

**Figure 5:**
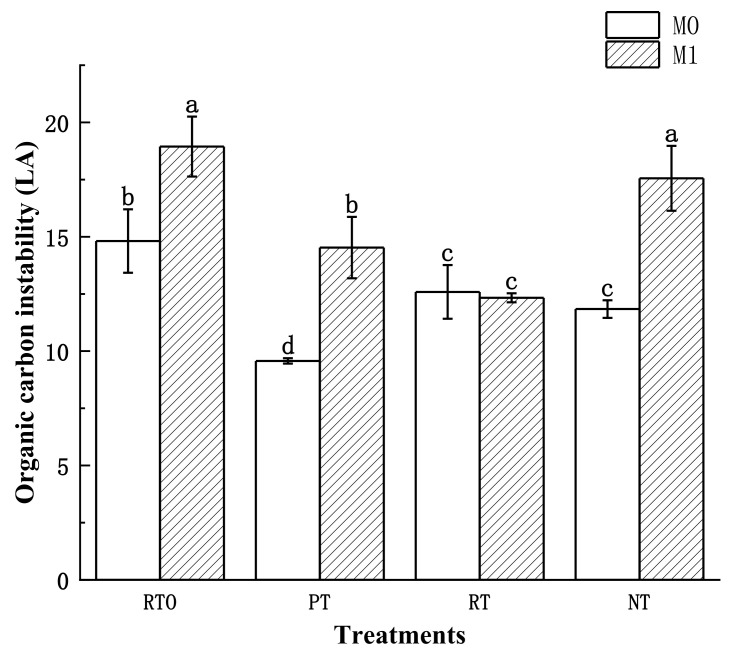
Different treatments and soil organic carbon instability. The error bars represent the standard deviation (SD). The significant differences between treatments are indicated by different lowercase letters labeled (*p* < 0.05, *n* = 3).

The results of the two-factor analysis of variance (Table 4) revealed that the soil LA was significantly influenced by the main effects of the soil tillage mode and straw addition level, as well as their significant interaction. After 70 days of cultivation, as depicted in Figure 5, the variation range of LA under different tillage and residue management practices ranged from 9.57 to 18.95. Without the straw addition, the trend in LA change among the treatments was RTO > RT > NT > PT. After adding straw, the trend shifted to RTO > NT > PT > RT. Except for the RT mode, the addition of straw significantly increased the LA in all treatments.

Compared to before cultivation, each treatment without the straw addition experienced a decrease in the LA. Among them, the PT mode showed the greatest decline, with a reduction of 61.57%. The NT and RT modes followed, with reductions of 38.10% and 15.18%, respectively. The RTO mode exhibited the smallest decline, with a reduction of 9.06%. In the treatments with the straw addition, all except the RTO mode experienced a decrease in the LA compared to before cultivation. The PT mode experienced the greatest decline, with a reduction of 41.68%. The RT mode followed with a decrease of 16.90%, and the NT mode exhibited the smallest decrease of 8.20%.

### 3.4. Impact Factor of Priming Effect

**Figure 6:**
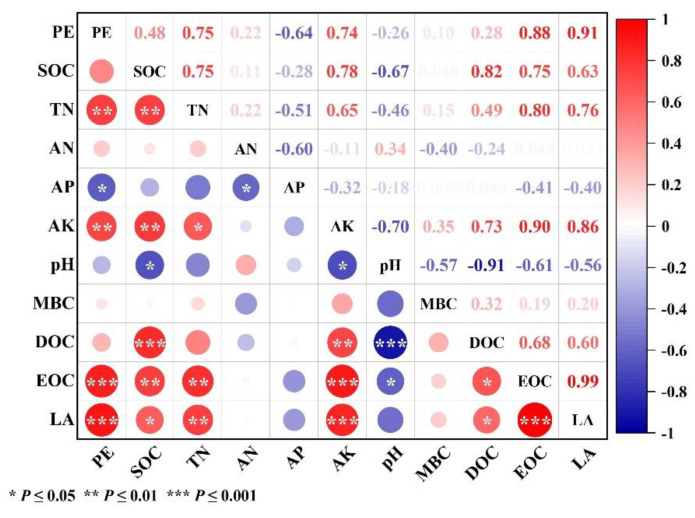
Correlation analysis between soil physicochemical property, apparent priming effect, and soil active organic carbon fractions (*p* < 0.05, *n* = 3).

According to Figure 6, it is evident that the soil LA is most strongly correlated with the soil priming effect, with a correlation coefficient of 0.906 (*p* < 0.01). Next, the soil EOC content shows the second-highest correlation. (r = 0.876, *p* < 0.01). It exhibits highly significant positive correlations with TN and AK, with correlation coefficients of 0.750 and 0.735, respectively (*p* < 0.01). Additionally, there is a significant negative correlation with AP (r = −0.638, *p* < 0.05). However, there is no significant correlation with the SOC, AN, pH, MBC, and DOC contents in the soil.

## 4. Discussion

In this study, after the addition of an equivalent amount of straw, the soil CO_2_ emission rates in each treatment exhibited distinct temporal variations that were characterized by a rapid decline phase, a fluctuating decline phase, and a mineralization stabilization phase. This mode aligns with the findings reported by Fontaine et al. and Thiessen et al. in their studies on the mineralization characteristics of soil organic carbon with the addition of different organic materials [40,41]. The observed phases may be attributed to the input of fresh organic materials, which provide additional carbon sources and nutrients, stimulating microorganisms to preferentially decompose and utilize labile carbon sources [42,43]. As the cultivation time extended, the easily decomposable organic matter in the soil was largely exhausted, and the remaining recalcitrant substances, such as cellulose and lignin, became less accessible for microbial utilization. This led to a suppression of microbial activity, and the rate of organic carbon mineralization tended to stabilize [44,45].

The variation in active organic carbon content among different treatments reveals their high sensitivity to tillage and straw management measures, confirming their suitability as indicators of soil organic matter and fertility impacts [46,47]. The addition of straw significantly increased the soil MBC, DOC, and EOC contents under different tillage modes. Simultaneously, this was accompanied by an increase in the soil organic carbon instability index. This could be attributed to the straw stimulating the decomposition of pre-existing organic carbon, enhancing organic carbon availability, promoting microbial growth, and subsequently increasing the soil MBC content—a viewpoint supported by previous studies [40]. Additionally, in addition, straw provides sufficient energy for soil microorganisms and promotes their reproduction and growth. [17]. *Gram-positive bacteria* and beneficial microbes, such as *Burkholderia*, participate in the transformation of soil organic carbon, promoting the decomposition of components like cellulose in straw. The sources of soil DOC include crop root exudates, crop residue degradation and translocation from straw cover, and microbial metabolites [48]. The PT mode might enhance the physical and biological decomposition of plant residues [49], leading to a higher DOC content compared to the other three tillage modes. In contrast, the RTO mode exhibited a significantly lower DOC content than the other three tillage modes, possibly due to the long-term absence of straw incorporation, resulting in relatively fewer microbial resources available. Therefore, the amount of DOC derived from crop residue degradation and microbial metabolic products is likely lower [50,51]. To a large extent, EOC seems to originate from the decomposition of newly added organic materials [47], aligning with the consistent findings of the present study where straw incorporation significantly increased the EOC content. In the RT mode, both the soil EOC content and the soil organic carbon instability index were relatively low, suggesting a lower organic matter decomposition rate and, consequently, a higher increase in organic carbon content in this tillage mode [49].

Abundant research indicates that the addition of exogenous organic carbon promotes microbial growth and increases soil extracellular enzyme activity, thereby stimulating the decomposition of pre-existing organic carbon in the soil, resulting in a positive priming effect [52]. In this study, the addition of straw under four different tillage modes led to increased CO_2_ emissions, enhanced the mineralization of soil organic carbon, and exhibited positive priming effects. Specifically, the addition of straw significantly increased the soil CO_2_ emission rates and the cumulative mineralization after 70 days of incubation in all treatments. On the one hand, this is due to the increase in reactive organic carbon content of the soil after incorporation of straw [53]. In this study, the addition of straw significantly increased the DOC and EOC contents in all treatments, and the mineralization of the active organic carbon fraction was a major contributor to soil respiration and CO_2_ emissions [21]. On the other hand, straw can serve as a carbon and nitrogen source for microbial metabolism, enhancing microbial activity and diversity and thereby promoting soil’s CO_2_ emissions [54,55]. This study identified significant differences in the intensity of the apparent priming effects in soils with straw incorporation under different tillage practices. Different tillage methods alter the soil’s physicochemical properties, which, in turn, change the structure and characteristics of the microbial community, resulting in differences in soil organic carbon mineralization and varying priming effect intensities [27,56]. The study also identified that different tillage practices with straw incorporation altered the physicochemical properties of the soil (Table 1) and confirmed that the intensity of soil priming effects is influenced by the soil’s physicochemical properties (Figure 6).

This study demonstrated that the sequence of stimulation effects across the three tillage and straw incorporation models was PT > NT > RT. Under the rotary tillage conditions, the impact of straw return on the priming effect was significant, with no straw return (RTO) exhibiting a markedly higher priming effect than straw return (RT).

The PT mode exhibited the strongest priming effect. This was attributed to the deep plowing in the PT mode, which effectively alleviated the restriction on crop root growth imposed by the plow pan layer compared to the other two tillage models [57]. This facilitated the growth, development, and increased return to the soil of roots after death, leading to elevated levels of easily decomposable simple organic carbon in the soil [43,58]. The significantly higher contents of SOC and active organic carbon (DOC, EOC, and LA) in the pre-cultivation PT mode, as presented in Table 1, strongly supported this observation. The addition of straw, along with the initially present easily decomposable simple carbon sources in the soil, provided a substantial initial energy supply for microorganisms, stimulating microbial decomposition activity. The results of this study indicated that, during the cultivation process, the PT mode exhibited the highest increase in DOC and MBC (Figure 3) contents when straw was added compared to when it was not added. The increments in EOC (Figure 4) and LA (Figure 5) were also relatively high. Therefore, in the early stages of cultivation, the CO_2_ emission rate in the PT mode (Figure 1) was significantly higher than in other treatments. Simultaneously, the combination of deep plowing and deep rotary tillage, in comparison to rotary tillage and no-tillage, subjected the soil to more frequent and extensive disturbances. This rendered the organic carbon more susceptible to microbial decomposition and utilization [49].

The NT mode significantly increased the MBC and EOC levels in the soil compared to RT. However, an elevated EOC content does not necessarily indicate decreased stability of the soil’s carbon pool [59]. Soil organic carbon stability likely depends more on physical protection, with active organic carbon shielded by macroaggregates, reducing susceptibility to microbial decomposition [60,61]. Under the NT mode, crop residues, primarily straw, tend to accumulate in the top 5 cm soil layer [62]. The carbon released from straw decomposition has a limited impact on the soil due to its low effectiveness. This results in the poorer structural stability of soil aggregates, causing the organic carbon protected by the aggregates to become more exposed and susceptible to microbial decomposition [63]. Research by Sarker et al. suggests that the increased availability of organically bound carbon within aggregates enhances soil organic carbon mineralization, intensifying the priming effect [23]. In RT, the priming effect is weaker with the straw incorporation compared to the RT soils without straw (RTO). The reduced effect may be attributed to the incorporation of straw into the soil, where it participates in the formation of soil aggregates as a binding material [64]. This process increases the proportion of large aggregates in the soil, providing stronger protection for the organic carbon components associated with the aggregates, ultimately reducing their biological availability and weakening the intensity of the priming effect [65].

We found no significant correlation between the priming effect and soil organic carbon (SOC) content, which is consistent with Dimassi’s findings [27]. We speculated that this lack of correlation could be attributed to the high C/N ratio of the straw added in this study, placing the soil in a relatively carbon-saturated state [66]. In such an environment, soil nitrogen became a limiting factor, favoring the dominance of K-strategist microorganisms. These microorganisms relied on the mineralization of soil organic matter to obtain additional nitrogen for growth and reproduction, a phenomenon referred to as the “nitrogen mining” theory [67,68]. While the nitrogen mineralization theory emphasizes the predominant role of microbial nitrogen limitation in the stimulation effect, microbial growth and reproduction were also dependent on other nutrient elements, particularly in regions where specific nutrient elements were limited [69,70]. This study further validated the significant or extremely significant correlations between the soil nitrogen, available phosphorus, available potassium, and the stimulation effect (Figure 6). Additionally, the stoichiometric decomposition theory posited that microbial activity was constrained by the most deficient nutrient element [71]. When the nutrient supply matched the microbial chemical stoichiometric ratio, microbial activity increased, leading to accelerated soil organic carbon mineralization and a greater likelihood of stimulation effects [72]. In the deep loosening model of this study, the soil’s nutrient availability was higher, aligning more closely with the nutrient chemical stoichiometric ratio required for microbial growth. Consequently, the stimulation effect was strongest in the deep loosening model, confirming the aforementioned viewpoint. Furthermore, this study indicated that the tillage intensity had a certain impact on the soil organic carbon pool components, especially unstable carbon fractions, making this portion of organic carbon more prone to stimulation [73]. This study revealed a highly significant positive correlation between the content of EOC in the soil and the instability of organic carbon concerning the priming effect (Figure 6). This finding provides a key explanation for the strongest priming effect observed in the PT model, followed by no-tillage with straw cover, conventional tillage without straw incorporation, and the weakest priming effect in the conventional tillage with straw incorporation model. This further corroborated Jenkinson’s notion that soils with highly labile organic carbon exhibited a strong positive priming effect [25].

## 5. Conclusions

The addition of straw promotes the mineralization of soil organic carbon, increases the content of active components and the instability of organic carbon, accelerates turnover, and enhances microbial activity in the soil. Among the different tillage practices, the deep loosening + deep rotary tillage straw incorporation mode exhibits the highest CO_2_ release and accumulation, with a relatively high content of active organic carbon components; in contrast, the rotary tillage straw incorporation mode shows the lowest CO_2_ release and accumulation. After the addition of straw, soils under various tillage practices demonstrated a positive stimulation effect, with the deep loosening + deep rotary tillage mode showing the strongest effect, followed by the no-tillage cover mode and the rotary tillage mode showing the weakest effect. However, the soil’s organic carbon content is highest under the rotary tillage mode. Analyses indicate that the apparent stimulation effect is significantly positively correlated with the unstable carbon components in the soil. Therefore, it is recommended that in the early 2–3 years of straw returning to the field in the Liao Nan region, the rotary tillage mode should be adopted to facilitate the formation and stabilization of organic carbon. As the time of straw return increases, the tillage practices should be adjusted based on the soil organic carbon sequestration and fertility conditions.

## Figures and Tables

**Table 1:** The properties and soil labile organic carbon fraction contents of soil samples tested before cultivation in 2020.

	RTO	RT	PT	NT
SOC (g·kg^−1^)	14.18 ± 0.17 c	14.49 ± 0.11 b	15.52 ± 0.04 a	13.75 ± 0.06 d
TN (g·kg^−1^)	1.65 ± 0.04 b	1.62 ± 0.03 b	1.74 ± 0.03 a	1.65 ± 0.03 b
AHN (mg·kg^−1^)	120.60 ± 0.76 a	112.08 ± 4.26 b	115.29 ± 6.22 ab	111.07 ± 2.65 b
AP (mg·kg^−1^)	27.34 ± 0.33 c	35.88 ± 0.68 a	28.08 ± 1.07 c	32.30 ± 0.50 b
AK (mg·kg^−1^)	178.28 ± 5.53 b	180.24 ± 2.21 b	212.88 ± 9.00 a	184.66 ± 6.63 b
MBC (mg·kg^−1^)	91.16 ± 6.11 c	98.48 ± 0.72 b	101.99 ± 10.08 ab	105.55 ± 9.54 a
DOC (mg·kg^−1^)	39.41 ± 1.75 d	47.91 ± 1.52 b	52.41 ± 0.65 a	42.47 ± 1.91 c
EOC (g·kg^−1^)	1.98 ± 0.13 c	1.87 ± 0.11 c	3.09 ± 0.16 a	2.21 ± 0.01 b
LA	16.29 ± 1.47 c	14.84 ± 1.11 c	24.91 ± 1.64 a	19.12 ± 0.08 b
pH	5.70 ± 0.07 a	5.35 ± 0.04 b	5.20 ± 0.01 c	5.46 ± 0.13 b

Notes: The significant differences between treatments are indicated by different lowercase letters labeled after the same column of data (*p* < 0.05). AHN = alkaline hydrolyzable nitrogen, AP = available phosphorus, AK = available potassium. MBC = soil active microbial carbon, DOC = dissolved organic carbon, EOC = easily oxidized organic carbon, and LA = soil organic carbon instability.

**Table 2:** Total organic carbon content of the soil under different treatments.

Time	Treatment	SOC
		(g·kg^−1^)
0 d	RTO	14.18 ± 0.17 c
	PT	15.52 ± 0.04 a
	RT	14.49 ± 0.11 b
	NT	13.75 ± 0.06 d
70 d	RTO + M0	15.04 ± 0.16 f
	RTO + M1	16.22 ± 0.12 c
	PT + M0	15.35 ± 0.09 e
	PT + M1	16.71 ± 0.16 b
	RT + M0	14.97 ± 0.08 f
	RT + M1	17.08 ± 0.09 a
	NT + M0	14.56 ± 0.01 g
	NT + M1	15.74 ± 0.06 d
Mode		***
Input		***
Mode × Input		***

Notes: The significant differences between treatments are indicated by different lowercase letters labeled. (*p* < 0.05, *n* = 3). “***” indicates that the *p*-values are significant at the 0.001 levels.

**Table 3:** Variance analysis of the effects of different treatments on soil active organic carbon content.

Target	MBC	DOC
Mode	***	***
Input	***	***
Time	***	***
Mode × Input	***	*
Mode × Time	***	**
Input × Time	***	*
Mode × Input × Time	***	***

Notes: “*”, “**”, and “***” indicate that the *p*-values are significant at the 0.05, 0.01, and 0.001 levels, respectively.

**Table 4:** Variance analysis of CO_2_ accumulative emission, SOC, EOC, and LA.

Target	Accumulation CO_2_ Emission	SOC	EOC	LA
Mode	***	***	***	***
Input	***	***	***	***
Mode × Input	***	***	**	***

Notes: “*”, “**” and “***” indicate that the *p*-values are significant at the 0.05, 0.01, and 0.001 levels, respectively.

## Data Availability

Data are contained within the article.
